# Prediction of the Clinical Severity of Progressive Supranuclear Palsy by Diffusion Tensor Imaging

**DOI:** 10.3390/jcm9010040

**Published:** 2019-12-24

**Authors:** Yao-Liang Chen, Xiang-An Zhao, Shu-Hang Ng, Chin-Song Lu, Yu-Chun Lin, Jur-Shan Cheng, Chih-Chien Tsai, Jiun-Jie Wang

**Affiliations:** 1 Department of Medical Imaging and Intervention, Chang Gung Memorial Hospital, Linkou 33305, Taiwan; Chenyl0702@adm.cgmh.org.tw (Y.-L.C.); shng@adm.cgmh.org.tw (S.-H.N.); jack805@gmail.com (Y.-C.L.); 2 Department of Medical Imaging and Intervention, Chang Gung Memorial Hospital, Keelung 20401, Taiwan; 3 Department of Medical Imaging and Radiological Sciences, Chang Gung University, Taoyuan 33302, Taiwan; samo011085@gmail.com; 4 Professor Lu Neurological Clinic, Taoyuan 33302, Taiwan; c81214@adm.cgmh.org.tw; 5 Division of Movement Disorders, Department of Neurology, Chang Gung Memorial Hospital, Linkou, Taoyuan 33378, Taiwan; 6 Neuroscience Research Center, Chang Gung Memorial Hospital, Linkou, Taoyuan 33302, Taiwan; 7 Clinical Informatics and Medical Statistics Research Center, Chang Gung University, Taoyuan 33302, Taiwan; jscheng@mail.cgu.edu.tw; 8 Department of Emergency Medicine, Chang Gung Memorial Hospital, Keelung 20401, Taiwan; 9 Healthy Aging Research Center, Chang Gung University, Taoyuan 33302, Taiwan; nasnas0218@gmail.com

**Keywords:** diffusion tensor imaging, progressive supranuclear palsy, UPDRS-III, LEDD, severity

## Abstract

Progressive supranuclear palsy (PSP) is characterized by a rapid and progressive clinical course. A timely and objective image-based evaluation of disease severity before standard clinical assessments might increase the diagnostic confidence of the neurologist. We sought to investigate whether features from diffusion tensor imaging of the entire brain with a machine learning algorithm, rather than a few pathogenically involved regions, may predict the clinical severity of PSP. Fifty-three patients who met the diagnostic criteria for probable PSP were subjected to diffusion tensor imaging. Of them, 15 underwent follow-up imaging. Clinical severity was assessed by the neurological examinations. Mean diffusivity and fractional anisotropy maps were spatially co-registered, normalized, and parcellated into 246 brain regions from the human Brainnetome atlas. The predictors of clinical severity from a stepwise linear regression model were determined after feature reduction by the least absolute shrinkage and selection operator. Performance estimates were obtained using bootstrapping, cross-validation, and through application of the model in the patients who underwent repeated imaging. The algorithm confidently predicts the clinical severity of PSP at the individual level (adjusted *R*^2^: 0.739 and 0.892, *p* < 0.001). The machine learning algorithm for selection of diffusion tensor imaging-based features is accurate in predicting motor subscale of unified Parkinson’s disease rating scale and postural instability and gait disturbance of PSP.

## 1. Introduction

Progressive supranuclear palsy (PSP) is one of the most common causes of neurodegenerative-parkinsonism after Parkinson’s disease (PD). Both share several similar clinical symptoms (bradykinesia, rigidity, dysarthria, dysphagia, and dementia), although resting tremor is rare in PSP [1]. Because of substantial overlaps in clinical symptoms and inadequate accuracy of current tests, differential diagnosis from PD is challenging [2]. PSP is generally characterized by a rapid and progressive clinical course. Therefore, an objective evaluation of disease severity at an early stage might significantly improve the diagnostic confidence of the neurologist.

Neuroimaging examination, such as structural magnetic resonance imaging (MRI) studies, is often prescribed on the suspected patients in order to rule out concomitant neurological disorders. Structural MRI-based signs can be detected in the brain of patients with PSP. For example, characteristic midbrain atrophy (“hummingbird” sign) on the midsagittal plane and rounded midbrain peduncles (“Mickey Mouse” sign) on the axial plane [3] were noticed. Unfortunately, they can only appear in advanced disease stages. Although they could be diagnostically useful, these measures or their ratios on the midbrain or pons were not found to be related to age, disease duration, or clinimetric scores and rarely result in meaningful change in patient management [4]. In recent years, diffusion tensor imaging (DTI) has been extensively used in the study of damages in white matter tracts [5]. Although DTI parameters, such as fractional anisotropy (FA) and mean diffusivity (MD), are deemed to reflect clinical rating systems, to be translated into clinical practice the measurements still need extensive method standardization [4].

From a pathological standpoint, PSP is a tauopathy characterized by diffuse deposits of globose neurofibrillary tangles, tufted astrocytes, and coiled bodies and threads in different brain areas [1]. The pathological alterations in PSP are not limited to the midbrain but do actually affect various regions including cerebellum, brainstem, deep nuclei, cerebral white and grey matter. Therefore, it might require a comprehensive assessment of the microstructural damage in the whole brain, in order to predict the clinical severity.

Previous radiomic studies on oncology already included multiple imaging features into a regression model for the prediction of treatment outcome [6,7]. Here, we designed a tailored machine learning algorithm in which the predictors of clinical severity were selected from a multivariable linear regression model. The aim is to examine if the metrics derived from DTI can be related to the severity of patients with PSP in order to support a timely clinical diagnosis.

## 2. Materials and Methods

This retrospective study is a re-analysis of images collected from three prospective studies during 2008–2017. All studies were reviewed and approved by the Institutional Review Board of Chang Gung Memorial Hospital (Approval No. 97-0510B, 98-3626A, 100-3761A3 and 201600426B0) and conducted following the Declaration of Helsinki. All participants provided written informed consent following a detailed explanation in the prospective studies.

### 2.1. Study Patients

All of the study patients were enrolled from the neurology clinics and had a clinical diagnosis of probable PSP according to the diagnostic criteria of either (1) National Institute of Neurological Disorders and Stroke (NINDS) and the Society for PSP (NINDS_PSP criteria, between July 2008 and August 2011) and (2) Litvan et al. [1] between June 2012 and December 2017. All the participants underwent MRI examinations using a 3T scanner. Both DTI and structural images (T1 weighted magnetization-prepared rapid acquisition gradient echo sequence, T1-MPRAGE) were acquired. Diagnoses of PSP were made by three senior neurologists (CS Lu, and YH Weng, and WY Lin, with 28, 21, and 8 years of experience, respectively). The following patients were excluded: presence of brain abnormalities including hydrocephalus or encephalomalacia that may impair cognitive function on MRI and/or ^18^fluorodeoxyglucose-positron emission tomography (^18^FDG-PET) studies; history of intracranial surgery such as thalamotomy, pallidotomy, and/or deep brain stimulation; and major physical or neuropsychiatric disorders; general MRI exclusion criteria. The study sample consisted of 53 patients (21 men and 32 women, mean age: 65.7 ± 6.5 years; mean disease duration: 5.4 ± 3.2 years). Of them, 15 patients (8 men and 7 women; mean age: 65.9 ± 5.7 years) underwent follow-up imaging examinations and served as an additional validation cohort.

Based on their clinical features [8], the study patients were divided into four different subgroups, as follows: (1) pure akinesia with gait freezing (PSP-PAGF), (2) parkinsonism (PSP-PD), (3) Richardson’s syndrome (PSP-RS), and (4) corticobasal syndrome (PSP-CBS). Results on the following clinical severity scales were obtained from all participants: (1) motor subscale of unified Parkinson’s disease rating scale (UPDRS-III), (2) postural impairment and gait disorder staging (PIGD) as the summary score of item 13, 14, 15, 29, 30 in the assessment of UPDRS, (3) modified Hoehn and Yahr staging (MHY) and (4) the intake of levodopa equivalent daily dose (LEDD). The general characteristics of the study participants are summarized in Table 1 and Supplementary Table S1 for the validation cohort.

### 2.2. Image Acquisition

Imaging was performed on a 3T MR scanner (Magnetom Trio; Siemens, Erlangen, Germany). A total of 160 contiguous axial T1-weighted images were acquired with T1-MPRAGE using the following parameters: TR/TE = 2000 ms/2.63 ms; flip angle = 9°; field of view = 224 mm × 256 mm, matrix size = 224 × 256–resulting in a voxel size of 1 mm × 1 mm × 1 mm. Three senior neuroradiologists (YL Chen, SH Ng, and YM Wu, with 28, 20, and 10 years of experience, respectively) blinded to clinical data independently interpreted all MR images. Diffusion images were acquired with three different imaging protocols. Two diffusion—weighting values (*b*-values)—0 and 1000 s/mm^2^—were used in the final analysis. Imaging parameters are shown in Table 1.

### 2.3. Image Processing

Images were processed as previously described by Ng and coworkers [9] using MATLAB (MATLAB 2015a; Math Works, Inc., Natick, MA, USA). Briefly, individual diffusion tensor parametric maps (MD and FA) were calculated from diffusion-weighted images with Diffusion Kurtosis Estimator software [10]. Using structural T1 images, a parenchymal mask was created to remove the signal from the cerebrospinal fluid. Both MD and FA maps were spatially co-registered, normalized, and parcellated into 210 cortical and 36 subcortical brain regions according to the Human Brainnetome Atlas [11] using the Statistical Parametric Mapping software (SPM8, 2009) [12]. The 90th, 50th, and 10th percentiles of each parcellated region of interest were recorded from diffusion tensor parametric maps, resulting in a total of 1476 features.

### 2.4. Statistical Analysis and Feature Reduction Process

All calculations were performed using the SAS statistical package, version 9.4 (SAS Institute Inc, Cary, NC, USA). The clinical severity scale was used as the ground truth and was entered into the regression model, which included UPDRS-III, PIGD, MHY and LEDD. All participants were used as the training cohort. The results were further validated using leave-one-out and five-fold cross-validation. In addition, the second imaging dataset from the 15 returned patients served as an additional blind validation data set.

The number of features was reduced in two procedures. First the L1-norm regularized least absolute shrinkage and selection operator (LASSO) regression with 1000 times bootstrapping was performed, in order to reduce the number of features to less than sample size (53, the number of the participants). To clarify the potential effect on the predictability of the models, age, sex, disease duration and imaging protocols were examined, together with the features from diffusion metric, by LASSO. The features which were selected by more than 20% of the bootstrapping models (i.e., ≥200 times) were entered into the second analysis. As a result, approximately 22 to 32 features from the original image features survived in each clinical severity scale.

Secondly, linear regression with stepwise selection was used to identify the features that were finally used to predict each clinical severity scale. The number of features that entered into each regression model is limited to one fifth of the participants’ number [13,14], which leads to 11 features in each regression model. The coefficient of these 11 features in the final model was determined by linear regression with 1000 times bootstrapping.

**Figure 1:**
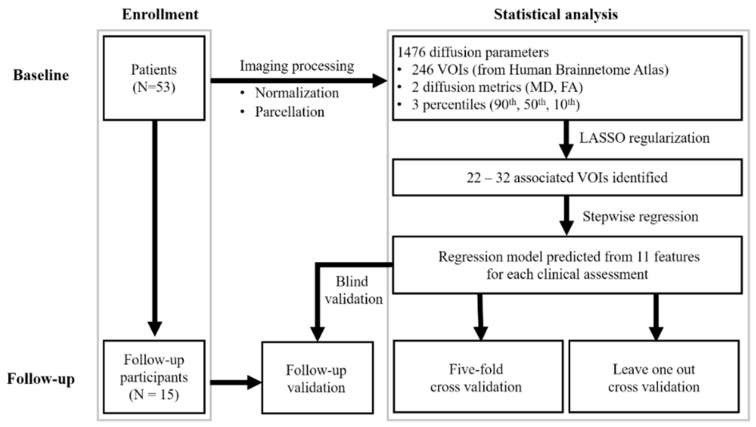
Flowchart of study design. VOI, volume of interest; UPDRS-III, motor subscale of Unified Parkinson’s Disease Rating Scale; PIGD, postural instability and gait disorder staging; MHY, modified Hoehn and Yahr staging; LEDD, levodopa equivalent daily dose.

The robustness of the regression model was expressed by the mean absolute error and the mean adjusted *R*^2^. We further validated our findings in the subgroup of patients who underwent serial MRI imaging (using the same image processing method). The extracted features were entered into the model developed in the initial analysis. For model PIGD, three missing data of the assessment mentioned in Table 1 were excluded in the analysis. The difference of mean absolute error among the cross validation in baseline (leave-one-out, five-fold) and the subgroup of patients was examined by using Friedman test with *p* < 0.05 was regarded as significant. Figure 1 is a flowchart of the study procedure.

## 3. Results

**Figure 2:**
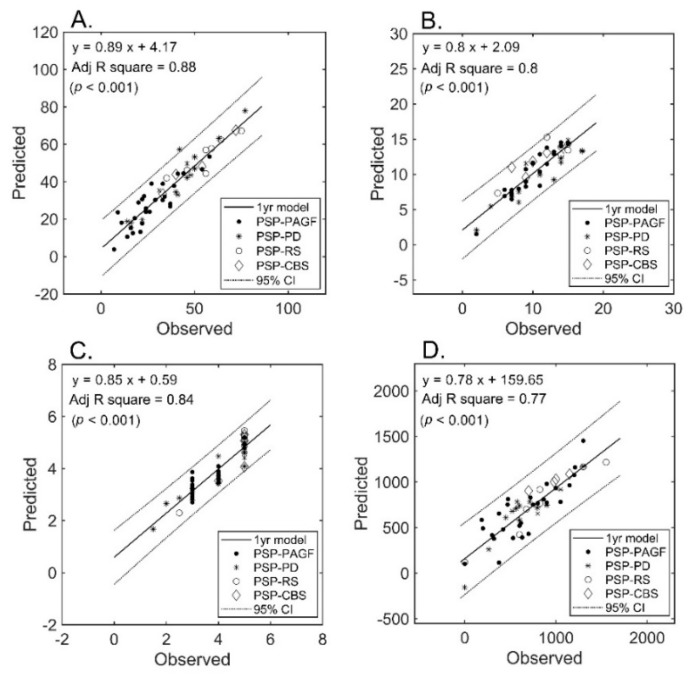
Prediction of severity measures: results of regression analysis. The graphs plot the observed versus predicted values for each severity measure at the individual level. (Panel (**A**)): UPDRS-III; (Panel (**B**)): PIGD; (Panel (**C**)): MHY; (Panel (**D**)): LEDD. UPDRS-III, motor subscale of Unified Parkinson’s Disease Rating Scale; PIGD, postural instability and gait disorder staging; MHY, modified Hoehn and Yahr staging; LEDD, levodopa equivalent daily dose.

The number of selected features after LASSO that were allowed to enter the regression model was inferior to the sample size (i.e., 32, 29, 24, and 22 for UPDRS-III, PIGD, MHY, and, LEDD respectively). Only diffusion metrics were selected into the final models. The final number of features in each prediction model was limited to 11 (approximately 53/5). The results of regression analysis revealed a strong correlation between diffusion imaging parameters and clinical severity (Figure 2) for all measures (A: UPDRS-III; B: PIGD; C: MHY; and D: LEDD). We were able to predict the clinical severity scores by using a combination of FA and MD values extracted from multiple regions of interest, i.e., not limited to areas known to be related to PSP pathogenesis. All of the predictions for the four different subtypes were within the 95% confidence interval, the only exception being UPDRS-III from one patient of PSP-PD (that fell shortly outside this interval).

**Figure 3:**
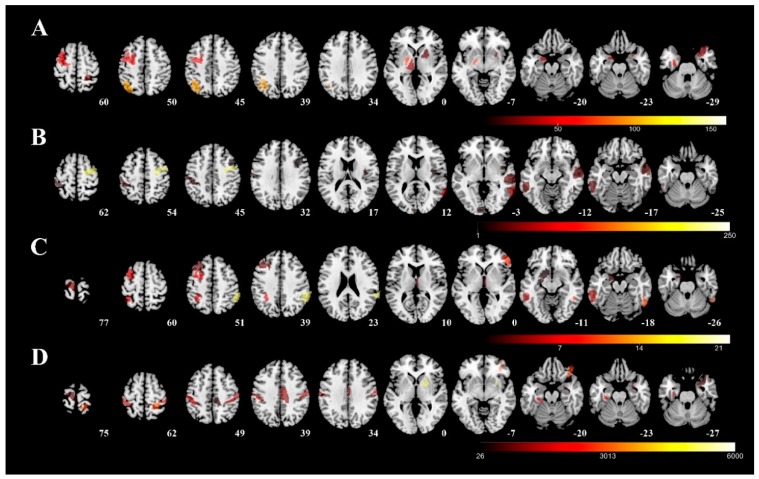
Predictive regions visualized using 3D rendering. The predictive regions for each severity measure were visualized using the unstandardized coefficient in the regression model and overlapped on a T1 template (average T1 scans obtained from 152 individuals examined in the Montreal Neurological Institute). Row (**A**): UPDRS-III; row (**B**): PIGD; row (**C**): MHY; row (**D**): LEDD. UPDRS-III, motor subscale of Unified Parkinson’s Disease Rating Scale; PIGD, postural instability and gait disorder staging; MHY, modified Hoehn and Yahr staging; LEDD, levodopa equivalent daily dose.

Figure 3 visualizes the involved regions for each severity tool (color representing the unstandardized coefficient in the regression model from each predictive region, row A: UPDRS-III; row B: PIGD; row C: MHY; row D: LEDD). The following five areas were identified as mainly associated with UPDRS-III: (1) left rostral area of parahippocampus; (2) caudal dorsolateral precentral gyrus; (3) globus pallidus; (4) sensory part of thalamus; and (5) right postcentral superior parietal lobule. The following five areas were found to be related to the PIGD: (1) dorsal granular part of insula; (2) dorsolateral area of middle temporal gyrus; (3) inferior frontal junction of middle temporal gyrus; (4) caudal dorsolateral area of precentral gyrus; and (5) inferior frontal sulcus of inferior temporal gyrus. As far as MHY is concerned, the following five areas were identified: (1) left ventrolateral area of middle frontal gyrus; (2) right caudal part of inferior parietal lobule; (3) left nucleus accumbens; (4) left lateral area of the superior parietal lobule; and (5) right rostral temporal part of thalamus. Finally, the following five areas of the right hemisphere were identified as mainly associated with LEDD: (1) right medial area of superior temporal gyrus; (2) right medial amygdala; (3) right caudal part of cingulate gyrus; (4) left posterior parahippocampus gyrus; and (5) right ventromedial putamen.

The predictive equations for each clinical assessment can be calculated in Table 2 by the combination of diffusion metrics from multiple brain regions and the unstandardized coefficients. Table 3 summarizes the statistical results of the regression model for each assessment at training and validation. In all assessments, the adjusted *R*^2^ varied from 0.739 to 0.892 in both model training and cross-validations. The mean absolute error of the estimation varied between 5.6% (UPDRS-III) and 40.1% (LEDD). The complete nomenclature-including diffusion metrics, percentile values, modified cytoarchitecture, and Montreal Neurological Institute (MNI) [15] coordinates are reported in Supplementary Table S2.

**Figure 4:**
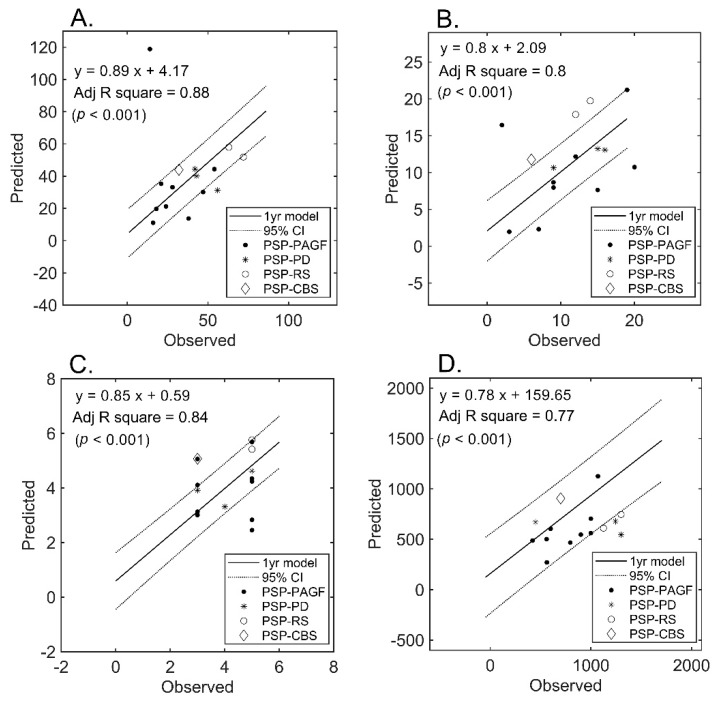
Prediction of severity measures: results of regression analysis in the validation subset. The graphs plot the observed versus predicted values for each severity measure at the individual level in the validation subset (*n* = 15). (Panel (**A**)): UPDRS-III; (Panel (**B**)): PIGD; (Panel (**C**)): MHY; (Panel (**D**)): LEDD. UPDRS-III, motor subscale of Unified Parkinson’s Disease Rating Scale; PIGD, postural instability and gait disorder staging; MHY, modified Hoehn and Yahr staging; LEDD, Levodopa equivalent daily dose.

Follow-up MRI examinations were performed in a subset of patients who served for blind validation purposes (Figure 4). An approximately less than three-fold increase in the estimation error was observed for all measures. However, the model initially developed in the entire cohort still retained its ability to predict UPDRS-III (mean absolute error: 15.5%) and, to a lesser extent, LEDD (mean absolute error: 33.9%). Figure 4 plots the predicted (using the initially developed model) versus observed scores in the subset of patients who underwent follow-up imaging.

## 4. Discussion

### 4.1. Main Findings

In this study, we developed a machine learning algorithm based on DTI to predict the clinical severity of PSP. Our model was found to be accurate for all of the clinical measures under consideration. Performance estimates of the prediction model were obtained using bootstrapping (1000 replications), leave-one-out/five-fold cross-validation, and through application of the model in the subset of patients who underwent repeated imaging. Notably the highest adjusted *R*^2^ was of 0.892 from UPDRS-III.

### 4.2. Clinical Impact

Despite continuing efforts, the identification of reliable imaging biomarkers for predicting PD severity remains elusive [16]. During the traditional diagnostic workout, MRI is generally performed to rule out concomitant neurological disorders. The use of our machine learning algorithm may allow a timely evaluation of disease severity before standard clinical assessments. This possibility may especially be advantageous in high-volume tertiary centers with long waiting lists. In this regard, traditional clinical evaluations in patients with movement disorders are known to be time-consuming and prone to fluctuations of symptoms over time.

In contrast, DTI data may be obtained rapidly (less than 15 min in our study) and are increasingly becoming a routine part of current MRI protocols. Notably, our data revealed a consistent association between microstructural damage reflected on DTI and clinical severity scales, most noticeably in the motor area (UPDRS-III and PIGD), even when the age, sex, disease duration and different imaging protocols were controlled. The rapid progression of the disease, assessed timely and accurately by our technique, might improve the diagnostic confidence of the neurologist, where an appropriate treatment course can be designed based on the response.

### 4.3. Regions Related to Motor Function

Basal ganglia are commonly considered as the main region involved in the pathogenesis of movement disorders. However, there is no obvious relation between the extent of damage in the basal ganglia and clinical severity. This observation is not unexpected given that the execution of movements requires inputs from multiple brain areas. Motor abnormalities occurring in patients with PSP are likely the results of an extensive involvement of various motor-related areas—including thalamus, precentral gyrus, middle temporal gyrus, and middle frontal gyrus.

In the current study, we found that DTI parameters measured in the thalamus were associated with UPDRS-III (sensory region) and MHY (rostral temporal part). Structural atrophy in the thalamus has been associated with an impaired motor function and seems to be one of the hallmarks to distinguish PSP from both PD and MSA [17]. Activation of these thalamic regions has been reported in pain, sleep, execution, attention, and noticeably-motion-related vision [18]. Similarly, the regions identified within globus pallidus and the sensory thalamus may be linked to the execution and/or planning of different action tasks [19]. Our data indicate that the severity of motor damage may be assessed by measuring the diffusion metric in multiple parcellated regions as selected from the whole brain.

### 4.4. Regions Related to Psychomotor Interactions

The clinical manifestations of PSP predominantly—but not exclusively—affect motor function. It is widely recognized that a proper motor execution requires adequate sensorimotor feedback, visuo-spatial perception, and motor learning. In the current study, the parahippocampus gyrus was found to contribute significantly to the prediction of both UPDRS-III and LEDD. This region is involved in memory and/or semantic language function [20], and its impairment has been related to memory decline in patients with PD [21]. A previous connectome analysis demonstrated numerous connections from the parahippocampus to subcortical regions—including thalamus, basal ganglia, hippocampus, and amygdala [11]. Besides its role in memory, the parahippocampus may therefore serve as a local functional hub linked to various operating nodes within the motor neural network. Our data may prompt further investigations into the role played by psychomotor functions in the clinical manifestations of PSP.

An accurate prediction of LEDD may be hampered by numerous factors that can influence drug dosage—including age, sex, disease duration, genetic background, and pathological status. Here, we found that regions associated with LEDD were related to face recognition (medial superior temporal gyrus) [22], emotions of fear or disgust (amygdala) [23], and emotion processing—especially in the reward/pain domain (caudal cingulate gyrus) [24]. Difficulties in recognizing negative emotions are part of the cognitive impairment occurring in patients with PSP [25], who are characterized by an impaired metabolism in this part of caudal cingulate gyrus [26]. The functions from our neuroimaging findings are in accordance with clinical observations showing apathy and impaired emotion processing of facial expressions in PSP [25]. The general prediction rule outlined in our study—based on a combination of cortical and subcortical neural networks—suggests that PSP is characterized by alterations in emotion and cognitive processing [27]. Consequently, a comprehensive evaluation of these patients cannot be limited to the sole assessment of motor function.

### 4.5. Validation of the Prediction Model

Notably, 15 of the 53 study patients performed a follow-up MRI examination and served as an additional validation cohort. Many conditions can contribute to the deviations in our prediction, for example, the patient condition at clinical evaluations, the disease courses or response to the treatment during the follow-up period, as well as the scanner fluctuation at acquisition and the subsequent post-processing procedures [28]. The difference in MAE of UPDRS III and LEDD obtained in this patient subset did not reach significance (*p* = 0.936 and 0.282, respectively) when compared with those from the cross validation analysis—suggesting that our prediction rule might be reliable and consistent in both assessments.

It can be observed that the prediction of LEDD in the validation cohort slightly deviated from the forecast-being characterized by the lowest adjusted *R*^2^ in the original model (leave-one-out/five-fold cross validation = 0.772 ± 0.008/0.739 ± 0.047). The response of patients with PSP to levodopa may be either absent or transient. However, there may be differences in the clinical spectrum of disease—with patients with PSP-RS showing poorer response than those with PSP-P [29]. The predominant inclusion of patients with PSP-P (as in our study) may lead to an underestimation of the predictive value. Notably, it has been previously suggested that common criteria for defining the response of patients with PSP to levodopa may not be entirely accurate and thus need further examination [30]. Similarly, our findings related to LEDD require additional validation in larger studies.

### 4.6. Technical Consideration and Additional Issues

Previous DTI analyses have been focused on white matter damage and frequently relied on complicated algorithms for accurate fiber tracking [31]—ultimately being unsuitable for routine clinical use. Notably, our current approach for predicting severity scores was not based on white matter tractography or connectivity analysis. We rather performed a reconstruction of the diffusion tensor followed by image normalization and parcellation—a method that can be easily implemented in a clinically-oriented environment with the use of freeware software SPM [12] or FSL (Functional magnetic resonance imaging of the brain Software Library [32]).

The inclusion of large amount of features might potentially result in model overfitting. To remove features unassociated with the outcome, several approaches have been developed, for example, principal component analysis [33] and independent component analysis [34]. However, it could be difficult to adjust the performance estimates when using these data-driven approaches [33,35]. The least absolute shrinkage and selection operator (LASSO) is a dimension-reduction technique which balances the bias and variance to minimize the mean squared error of the predictive model [36]. Features survived after LASSO procedure has been shown to be stable in the predictive performance when compared with other feature selection methods [37]. Here, we implemented a L1-norm regularized LASSO procedure to reduce the number of features that could be entered into the regression model. The result showed that the number of the features survived LASSO (22 to 32 for different clinimetrics) was less than the sample size (53 patients). The final number of the features in each predictive model was further reduced to 1/5 of the sample size at stepwise regression. This procedure was believed to be able to minimize the identification of spurious correlations [36].

PSP was a neurodegenerative disease caused by four-repeat (4R) tauopathy and region-specific tau deposits. It was postulated that the increase of amyloid-β (Aβ) might trigger tau pathology leading to the eventual neuronal death [38]. However, an appropriate tau-ligand positron emission tomography (PET) seems yet to be developed. Although the ATN characterization (biomarker of Ab-amyloid, Tau and Neurodegeneration or Neuronal injury) and of post-mortem evidence are not available to our study, it is less likely that these PSP patients could be amyloid positive according to the different clinical presentations, underlying neuropathological findings and our DTI imaging characteristics.

Because the subgroups of PSP patients was considered as different clinical presentations which might be related to region-specific tau deposits [39], it would require increasing number of patients in each subgroup to reliably verify our result. The combination DTI with appropriate tau-PET might be a powerful and complimentary approach to give precise diagnosis and prognosis of PSP. In the future study, it would be interesting to investigate this disease with specific radioactive tracers that is appropriate and clinically available to the general practice.

### 4.7. Limitations

Because of the retrospective nature of the study, the specific scale of PSP assessment is not available and the images of the participants were collected from three MRI imaging protocols, in an effort to increase the number of participants. However, the UPDRS-III and PIGD are used for the general evaluation of the clinical severity. Our result showed that the predictive models can be valid for different imaging protocols.

Owing to the data-driven approach, we cannot infer any direct causal relationship between the observed brain alterations and clinical severity measures. The question as to whether these associations are truly causal needs to be addressed in larger longitudinal investigations.

Because PSP is a rare disease, we were only able to include 53 patients in our analysis. Therefore, we did not divide a specific portion of the participants as a k-folds hold-out validation cohort. However, we did used a subset of data from 15 returned patients as an additional validation. A methodological point that merits comment is that diffusion metrics in volumes of interest are generally reported as means. It is difficult to forecast whether such parameters would increase (or decrease) under certain disease states. Consequently, the 90th, 50th, and 10th percentiles of each variable were recorded in this study. Being unrelated to the morphometric features of the ROI (e.g., length, area, and volume)—these values contributed to the accuracy of the normalization algorithm.

## Figures and Tables

**Table 1:** General characteristics of the study patients and clinical scores of progressive supranuclear palsy (PSP) patients.

	Protocol A	Protocol B	Protocol C	Total
TE/TR (ms)	83/7800	96/8200	108/5700	
Voxel size	2 × 2 × 2	2 × 2 × 2	2 × 2 × 3	
Directions	64	64	30	
PSP				
Number of patients	19	11	23	53
Sex (men/women)	7/12	6/5	8/15	21/32
Age (years)	63.9 ± 6.0	64.2 ± 6.6	67.8 ± 6.5	65.7 ± 6.5
Disease duration (years)	5.6 ± 2.3	4.2 ± 2.6	5.9 ± 3.9	5.4 ± 3.2
Subtype (PAGF/PD/RS/CBS)	5/8/5/1	7/4/0/0	15/3/2/3	27/15/7/4
UPDRS-III (motor)	29.6 ± 13.9 ^#^	45.8 ± 17.0	32.0 ± 17.3	36.5 ± 17.7
PIGD	10.8 ± 4.1 (NA = 3)	9.6 ± 4.1	10.8 ± 3.3	10.5 ± 3.7 (NA = 3)
MHY	4.0 ± 1.1	3.7 ± 1.1	3.8 ± 0.9	3.9 ± 1.0
<3	2	1	1	4
3	5	4	9	18
4	3	3	6	12
5	9	3	7	19
LEDD (mg/day)	708.9 ± 311.8	615.0 ± 253.6	758.3 ± 426.6	724.5 ± 343.9

Data are presented as counts or means ± standard deviations, as appropriate. Protocol A was used from July 2008 to April 2010; protocol B from January 2010 to August 2011; protocol C from June 2012 to December 2017. Abbreviations: TR, repetition time; TE, echo time; PAGF, pure akinesia with gait freezing; PD, Parkinson’s disease; RS, Richardson’s syndrome; CBS, corticobasal syndrome; UPDRS-III, motor subscale of Unified Parkinson’s Disease Rating Scale; PIGD, postural instability and gait disorder staging; MHY, modified Hoehn and Yahr staging; LEDD, levodopa equivalent daily dose; # indicates significant differences between protocol A and protocol C (*p* = 0.01). NA, Not available.

**Table 2:** Predictive variables in regression model for each assessment.

	UPDRS-III =		PIGD =		MHY =		LEDD =
−	100.6	+	1.2	+	6.0	+	450.9
+	48.7 × MD50_PhG_L_6_1	+	1.5 × MD90_INS_R_6_5	−	2.8 × MD50_MFG_L_7_5	+	2833.7 × FA90_STG_R_6_1
+	51.2 × MD10_PrG_L_6_2	+	210.7 × FA10_MTG_R_4_3	−	6.7 × FA90_IPL_R_6_4	−	571.6 × MD50_Amyg_R_2_2
+	28.3 × FA90_GP_L	−	49.9 × FA90_MTG_R_4_3	+	9.5 × FA50_NAC_L	+	325.4 × MD50_CG_R_7_6
+	65.2 × MD10_Tha_L_8_3	−	26.0 × FA90_MFG_R_7_2	−	3.8 × FA90_SPL_L_5_3	−	369.4 × MD90_PhG_L_6_3
−	23.9 × FA90_SPL_R_5_4	+	4.1 × MD90_PrG_R_6_2	−	10.5 × FA90_Tha_R_8_4	−	2074.1 × FA10_VM_Put_R
+	98.2 × FA90_STG_R_6_1	+	28.4 × FA50_ITG_R_7_2	−	4.9 × MD10_IFG_R_6_4	+	2949.2 × FA50_OrG_R_6_2
−	35.9 × MD10_Amyg_L_2_1	−	13.7 × FA90_MTG_R_4_4	+	6.1 × FA50_ITG_R_7_2	+	1487.9 × MD10_PrG_L_6_4
+	72.8 × MD10_Tha_L_8_8	+	9.3 × FA90_PoG_L_4_3	+	18.6 × FA10_ITG_L_7_6	−	1568.8 × MD10_PoG_L_4_3
−	18.0 × MD90_IPL_L_6_2	−	5.5 × MD10_Amyg_R_2_1	+	2.0 × MD10_PrG_L_6_4	−	1112.0 × FA90_PCL_R_2_1
+	35.0 × FA90_VM_Put_R	+	3.7 × MD90_MVOcC_L_5_3	−	0.6 × MD50_Amyg_L_2_1	+	421.3 × MD50_PoG_R_4_3
−	38.1 × FA90_MFG_L_7_6	+	44.6 × FA10_ITG_L_7_6	−	2.6 × FA90_MFG_L_7_6	+	5248.2 × FA10_SPL_R_5_4

The table illustrates the predictive variables and the corresponding unstandardized coefficients for each assessment. UPDRS-III, motor subscale of unified Parkinson’s disease rating scale; PIGD, postural instability and gait disorder staging; MHY, modified Hoehn and Yahr staging; LEDD, levodopa equivalent daily dose. MD, mean diffusivity; FA, fractional anisotropy; Dependent variables were as follows: STG, superior temporal gyrus; Amyg, amygdala gyrus; CG, cingulate gyrus; PhG, parahippocampal gyrus; VM_Put, ventromedial putamen; OrG, orbital gyrus; PrG, precentral gyrus; PoG, postcentral gyrus; PCL, paracentral lobule; SPL, superior parietal lobule; GP, globus pallidus; Tha, thalamus; IPL, inferior parietal lobule; MFG, middle frontal gyrus; NAC, nucleus accumbens; ITG, inferior temporal gyrus; INS, insular gyrus; MTG, middle temporal gyrus; MVOcC, msedioventral occipital cortex.

**Table 3:** Statistics of regression model for each assessment.

	UPDRS-III	PIGD	MHY	LEDD
Training				
Adjusted *R*^2^ (95% CI)	0.88 (0.83~0.93)	0.80 (0.72~0.88)	0.85 (0.79~0.91)	0.77 (0.69~0.85)
F Test	395	194	284	176
Cohen f^2^	3.43	1.78	2.60	1.46
Power	1.00	1.00	1.00	1.00
LOOCV				
Mean Adjusted *R*^2^	0.884 ± 0.005	0.799 ± 0.010	0.845 ± 0.006	0.772 ± 0.008
MAE	6.1 ± 5.0	1.7 ± 1.6	0.4 ± 0.3	180.8 ± 119.9
MAE in %	5.6 ± 4.6	8.2 ± 7.8	8.0 ± 5.8	32.9 ± 42.6
Five-fold CV				
Mean Adjusted *R*^2^	0.892 ± 0.016	0.818 ± 0.033	0.856 ± 0.024	0.739 ± 0.047
MAE	6.37 ± 0.89	1.845 ± 0.689	0.413 ± 0.056	223.0 ± 48.2
MAE in %	5.9 ± 0.8	9.2 ± 3.4	8.2 ± 1.1	40.1 ± 8.2
Follow-up Validation MAE	16.8 ± 25.6 ^a^	4.3 ± 3.9 ^b^	1.0 ± 0.8 ^c^	313.8 ± 220.6 ^d^
MAE in %	15.5 ± 23.7	21.4 ± 19.7	20.5 ± 16.0	33.9 ± 17.9

Adjusted *R*^2^ and F values are reported at the end of each regression model. UPDRS-III, motor subscale of unified Parkinson’s disease rating scale; PIGD, postural instability and gait disorder staging; MHY, modified Hoehn and Yahr staging; LEDD, levodopa equivalent daily dose in unit of mg/day. The averaged adjusted *R*^2^ and mean average errors between forecasted and observed values were obtained from leave-one-out/five-fold validation. Abbreviations: CI, confidence interval; MAE, mean absolute error; LOOCV, leave one out cross validation; Five-fold CV, five-fold cross validation. Reported adjusted *R*^2^ were all significant (*p* < 0.001). Comparison of MAE among LOOCV, five-fold CV and follow-up validation in subject who returned for this study. ^a^, *p* = 0.936; ^b^, *p* = 0.001; ^c^, *p* = 0.006; ^d^, *p* = 0.282.

## Supplementary Materials

The following are available online at https://www.mdpi.com/2077-0383/9/1/40/s1, Table S1: general characteristics of patients included in the validation cohort. Table S2: nomenclature of the features for each assessment in the predictive model.
