# Supplementary material for: Prediction of the Clinical Severity of Progressive Supranuclear Palsy by Diffusion Tensor Imaging
Source: J Clin Med. 2019 Dec 24;9(1):40. doi: 10.3390/jcm9010040 (PMC7020078; doi:10.3390/jcm9010040)
Supplement: Supplementary file 1 [file jcm-09-00040-s001.pdf]

Supplementary Materials

Supplementary Table S1. General characteristics of patients included in the validation cohort.

	Protocol A	Protocol B	Protocol C	Total
TE / TR (ms)	83 / 7800	96 / 8200	108 / 5700	
Voxel size	2 × 2 × 2	2 × 2 × 2	2 × 2 × 3	
Directions	64	64	30	
Number of patients	8	4	3	
Sex (men/women)	4 / 4	2 / 2	2 / 1	8 / 7
Age (years)	65.1 ± 4.1	62.5 ± 5.8	72.3 ± 5.5	65.9 ± 5.7
Disease duration (years)	8.0 ± 2.4	6.5 ± 3.5	7.7 ± 2.1	7.5 ± 2.6
Subtype (PAGF/PD/RS/CBS)	3 / 2 / 2 / 1	3 / 1 / 0 / 0	3 / 0 / 0 / 0	9 / 3 / 2 / 1
UPDRS-III (motor)	45.5 ± 18.4	26.5 ± 11.3	32.0 ± 17.3	37.9 ± 18.1
PIGD	12.0 ± 4.7	7.3 ± 6.0	10.8 ± 3.3	11.2 ± 5.4
MHY	4.3 ± 1.0	3.8 ± 1.0	3.8 ± 0.9	4.1 ± 1.0
<3	0	0	0	0
3	3	2	1	6
4	0	1	0	1
5	5	1	2	8
LEDD (mg/day)	921.9 ± 318.3	917.5 ± 301.8	660.8 ± 302.2	868.5 ± 307.9

Data are presented as counts or means ± standard deviations, as appropriate. Protocol A was used from July 2008 to April 2010; protocol B from Jun 2011 to Nov 2011; protocol C from Sep 2013 to Nov 2017. Data on age and disease and the results of clinical assessments were collected at baseline. Abbreviations: TR, repetition time; TE, echo time; UPDRS-III, motor subscale of Unified Parkinson's Disease Rating Scale; PIGD, postural instability and gait disorder staging; MHY, modified Hoehn and Yahr staging; LEDD, levodopa equivalent daily dose.

Supplementary Table S2. Nomenclature of the features for each assessment in the predictive model.

UPDRS-III:	Modified cytoarchitectonic	MNI (X, Y, Z)
MD50_PhG_L_6_1: Para-hippocampal gyrus (Left side)	A35/36r, rostral area 35/36	(−27, −7, −34)
MD10_PrG_L_6_2: Precentral gyrus (Left side)	A6cdl, caudal dorsolateral area 6	(−32, −9, 58)
FA90_GP_L: globus pallidus (Left side)	GP, globus pallidus	(−22, −2, 4)
MD10_Tha_L_8_3: Thalamus (Left side)	Stha, sensory thalamus	(−18, −23, 4)
FA90_SPL_R_5_4: Superior parietal lobule (Right side)	A7pc, postcentral area 7	(23, −43, 67)
FA90_STG_R_6_1: Superior temporal gyrus (Right side)	A38m, medial area 38	(31, 15, −34)
MD10_Amyg_L_2_1: Amygdala (Left side)	mAmyg, medial amygdala	(−19, −2, −20)
MD10_Tha_L_8_8: Thalamus (Left side)	lPFtha, lateral pre-frontal thalamus	(−11, −14, 2)
MD90_IPL_L_6_2: Inferior parietal lobule (Left side)	A39rd, rostrodorsal area 39 (Hip3)	(−38, −61, 46)
FA90_VM_Put_R: Ventromedial putamen (Right side)	vmPu, ventromedial putamen	(22, 8, −1)
FA90_MFG_L_7_6: Middle frontal gyrus (Left side)	A6vl, ventrolateral area 6	(−32, 4, 55)
**PIGD:**		
MD90_INS_R_6_5: Insular gyrus (Right side)	dIg, dorsal granular insula	(39, −7, 8)
FA10_MTG_R_4_3: Middle temporal gyrus (Right side)	A37dl, dorsolateral area 37	(60, −53, 3)
FA90_MFG_R_7_2: Middle frontal gyrus (Right side)	IFJ, inferior frontal junction	(42, 11, 39)
MD90_PrG_R_6_2: Precentral gyrus (Right side)	A6cdl, caudal dorsolateral area 6	(33, −7, 57)
FA50_ITG_R_7_2: Inferior temporal gyrus (Right side)	A37elv, extreme lateroventral area 37	(48, 35, 13)
FA90_MTG_R_4_4: Middle temporal gyrus (Right side)	aSTS, anterior superior temporal sulcus	(58, −16, −10)
FA90_PoG_L_4_3: Postcentral gyrus (Left side)	A2, area 2	(−46, −30, 50)
MD10_Amyg_R_2_1: Amygdala (Right side)	mAmyg, medial amygdala	(19, −2, −19)
MD90_MVOcC_L_5_3: Medioventral occipital cortex (Left side)	cCunG, caudal cuneus gyrus	(−6, −94, 1)
FA10_ITG_L_7_6: Inferior temporal gyrus (Left side)	A20cl, caudolateral of area 20	(−59, −42, −16)
**MHY:**		
MD50_MFG_L_7_5: Middle frontal gyrus (Left side)	A8vl, ventrolateral area 8	(−33, 23, 45)
FA90_IPL_R_6_4: Inferior parietal lobule (Right side)	A40c, caudal area 40 (PFm)	(57, −44, 38)
FA50_NAC_L: Nucleus accumbens (Left side)	NAC, nucleus accumbens	(−17, 3, −9)
FA90_SPL_L_5_3: Superior parietal lobule (Left side)	A5l, lateral area 5	(−33, −47, 50)
FA90_Tha_R_8_4: Thalamus (Right side)	rTtha, rostral temporal thalamus	(3, −13, 5)
MD10_IFG_R_6_4: Inferior frontal gyrus (Right side)	A45r, rostral area 45	(51, 36, −1)
FA50_ITG_R_7_2: Inferior temporal gyrus (Right side)	A37elv, extreme lateroventral area 37	(53, −52, −18)
FA10_ITG_L_7_6: Inferior temporal gyrus (Left side)	A20cl, caudolateral of area 20	(−59, −42, −16)
MD10_PrG_L_6_4: Precentral gyrus (Left side)	A4t, area 4 (trunk region)	(−13, −20, 73)
MD50_Amyg_L_2_1: Amygdala (Left side)	mAmyg, medial amygdala	(−19, −2, −20)
FA90_MFG_L_7_6: Middle frontal gyrus (Left side)	A6vl, ventrolateral area 6	−32, 4, 55
**LEDD:**		
FA90_STG_R_6_1: Superior temporal gyrus (Right side)	A38m, medial area 38	(31, 15, −34)
MD50_Amyg_R_2_2: Amygdala (Right side)	mAmyg, medial amygdala	(28, −3, −20)
MD50_CG_R_7_6: Cingulate gyrus (Right side)	A23c, caudal area 24	(6, −20, 40)
MD90_PhG_L_6_3: Para-hippocampal gyrus (Left side)	lateral PPHC, posterior parahippocampal gyrus	(−28, −32, −18)
FA10_VM_Put_R: Ventromedial putamen (Right side)	vmPu, ventromedial putamen	(22, 8, −1)
FA50_OrG_R_6_2: Orbital gyrus (Right side)	A12/47o, orbital area 12/47	(40, 39, −14)
MD10_PrG_L_6_4: Precentral gyrus (Left side)	A4t, area 4 (trunk region)	(−13, −20, 73)
MD10_PoG_L_4_3: Postcentral gyrus (Left side)	A2, area 2	(−46, −30, 50)
FA90_PCL_R_2_1: Paracentral lobule (Right side)	A1/2/3ll, area 1/2/3 (lower limb region)	(10, −34, 54)
MD50_PoG_R_4_3: Postcentral gyrus (Right side)	A2, area 2	(48, −24, 48)
FA10_SPL_R_5_4: Superior parietal lobule (Right side)	A7pc, postcentral area 7	(23, −43, 67)

The nomenclature – including the diffusion index, the percentile values, as well as the modified cytoarchitectonic and Montreal Neurological Institute (MNI) coordinates – for each assessment in the predictive model was extracted from Fan Lingzhong *et al*. (2016). Abbreviations: MD, mean diffusivity; FA, fractional anisotropy; _90, 90th percentile of the maximum value in the VOI; _50, 50th percentile of the median value in the VOI; _10, 10th percentile of the minimum value in the VOI. UPDRS-III, motor subscale of Unified Parkinson's Disease Rating Scale; PIGD, postural instability and gait disorder staging; MHY, modified Hoehn and Yahr staging; LEDD, levodopa equivalent daily dose.
